# A Manual of Modern Surgery

**Published:** 1895-06

**Authors:** 


*A Manual of Modern Surgery*. By JOHN CHALMERS DACOSTA, M.D. Pp. 809. Philadelphia: W. B. SAUNDERS. 1894.

This book may be safely recommended to students as a handy and trustworthy manual of modern surgery. It does not profess to contain original work, but aims at giving in clear terms and in concise form the fundamental principles of surgery. Great care has been taken to keep the work within a small compass, consequently ophthalmology, otology, and gynæcology are omitted; but considerable space is devoted to fractures and dislocations, while operative surgery receives a large share of attention. We have been especially pleased with the section devoted to abdominal surgery; important subjects like appendicitis, enterectomy, the radical cure of hernia, cystotomy, &c. being well and plainly dealt with. The chapters on bacteriology, inflammation, and repair are written from a modern standpoint, and are worthy of the highest praise, inasmuch as they are calculated to attract rather than repel the early student of surgery who has so often turned away with repugnance from this threshold of his subject. We know of no other *small* work on surgery in the English language which so well fulfils the requirements of the modern student.

